# Direct oral anticoagulants for the treatment of cerebral venous thrombosis – a protocol of an international phase IV study

**DOI:** 10.3389/fneur.2023.1251581

**Published:** 2023-09-14

**Authors:** Anita van de Munckhof, Mayte Sánchez van Kammen, Katarzyna Krzywicka, Sanjith Aaron, Diana Aguiar de Sousa, Florina Antochi, Antonio Arauz, Miguel A. Barboza, Adriana B. Conforto, Francesco Dentali, Daniel Galdames Contreras, Xunming Ji, Katarina Jood, Mirjam R. Heldner, María Hernández-Pérez, Wayneho Kam, Timothy J. Kleinig, Espen S. Kristoffersen, Ronen R. Leker, Robin Lemmens, Sven Poli, Nilüfer Yeşilot, Mohammad Wasay, Teddy Y. Wu, Marcel Arnold, Lia Lucas-Neto, Saskia Middeldorp, Jukka Putaala, Turgut Tatlisumak, José M. Ferro, Jonathan M. Coutinho

**Affiliations:** ^1^Department of Neurology, Amsterdam UMC, Location University of Amsterdam, Amsterdam, Netherlands; ^2^Department of Neurology, Christian Medical College, Vellore, India; ^3^Department of Neurology, Stroke Center, Centro Hospitalar Universitário de Lisboa Central, Lisbon, Portugal; ^4^Department of Neurology, Spitalul Universitar de Urgenţă Bucureşti, Bucharest, Romania; ^5^Department of Neurology, National Institute of Neurology and Neurosurgery, Mexico City, Mexico; ^6^Department of Neurology, Rafael Angel Calderon Guardia Hospital, San José, Costa Rica; ^7^Department of Neurology, Hospital das Clínicas da Faculdade de Medicina da Universidade de São Paulo, São Paulo, Brazil; ^8^Department of Neurology, Asst Sette Laghi, Varese, Italy; ^9^Stroke Unit, Hospital Clínico de la Universidad de Chile, Santiago, Chile; ^10^Department of Neurology, Xuanwu Hospital, Capital Medical University, Beijing, China; ^11^Department of Neurology, Sahlgrenska University Hospital, Gothenburg, Sweden; ^12^Department of Clinical Neuroscience, Institute of Neuroscience and Physiology, Sahlgrenska Academy at University of Gothenburg, Gothenburg, Sweden; ^13^Department of Neurology, Inselspital, University Hospital and University of Bern, Bern, Switzerland; ^14^Department of Neurology, Hospital Germans Trias i Pujol, Badalona, Spain; ^15^Department of Neurology, Duke University Hospital, Durham, NC, United States; ^16^Department of Neurology, Royal Adelaide Hospital, Adelaide, SA, Australia; ^17^Department of Neurology, Akershus University Hospital, Nordbyhagen, Norway; ^18^Department of Neurology, Hadassah – Hebrew University Medical Center, Jerusalem, Israel; ^19^Department of Neurology, UZ Leuven, Leuven, Belgium; ^20^Department of Neurology, Tübingen University Hospital, Tübingen, Germany; ^21^Department of Neurology, Istanbul Tip Fakültesi, Istanbul, Turkey; ^22^Department of Neurology, Aga Khan University, Karachi, Pakistan; ^23^Department of Neurology, Christchurch Hospital, Christchurch, New Zealand; ^24^Department of Neuroradiology, Centro Hospitalar Universitário Lisboa Norte, Lisbon, Portugal; ^25^Department of Internal Medicine, Radboud University Medical Center, Nijmegen, Netherlands; ^26^Department of Neurology, Helsinki University Hospital and University of Helsinki, Helsinki, Finland; ^27^Centro de Estudos Egas Moniz, Faculdade de Medicina, Universidade de Lisboa, Lisbon, Portugal

**Keywords:** cerebral venous thrombosis, anticoagulants, DOAC, vitamin K antagonist, treatment

## Abstract

**Introduction:**

Current guidelines recommend that patients with cerebral venous thrombosis (CVT) should be treated with vitamin K antagonists (VKAs) for 3–12 months. Direct oral anticoagulants (DOACs), however, are increasingly used in clinical practice. An exploratory randomized controlled trial including 120 patients with CVT suggested that the efficacy and safety profile of dabigatran (a DOAC) is similar to VKAs for the treatment of CVT, but large-scale prospective studies from a real-world setting are lacking.

**Methods:**

DOAC-CVT is an international, prospective, observational cohort study comparing DOACs to VKAs for the prevention of recurrent venous thrombotic events after acute CVT. Patients are eligible if they are 18 years or older, have a radiologically confirmed CVT, and have started oral anticoagulant treatment (DOAC or VKA) within 30 days of CVT diagnosis. Patients with an absolute contra-indication for DOACs, such as pregnancy or severe renal insufficiency, are excluded from the study. We aim to recruit at least 500 patients within a three-year recruitment period. The primary endpoint is a composite of recurrent venous thrombosis and major bleeding at 6 months of follow-up. We will calculate an adjusted odds ratio for the primary endpoint using propensity score inverse probability treatment weighting.

**Discussion:**

DOAC-CVT will provide real-world data on the comparative efficacy and safety of DOACs versus VKAs for the treatment of CVT.

**Clinical trial registration:**

ClinicalTrials.gov, NCT04660747.

## 1. Introduction

Cerebral venous thrombosis (CVT) is a rare thrombotic disorder that mainly affects adult women (1, 2). The clinical manifestations of CVT vary per patient, and can include severe headache, neurological deficits, epileptic seizures, and coma (3). Both the European and American CVT guidelines recommend treatment with (low-molecular weight) heparins in the acute phase, followed by vitamin K antagonists (VKAs) for a period of 3–12 months to prevent recurrent venous thrombotic events (VTEs), including new CVT (4, 5).

For the acute treatment and secondary prevention of VTEs other than CVT, such as lower extremity deep-vein thrombosis and pulmonary embolism, direct oral anticoagulants (DOACs) have largely replaced VKAs as first-line therapy because of a similar efficacy and lower bleeding risk. In these conditions, DOACs carry a lower risk of intracranial hemorrhage compared to VKAs (6). In addition to a superior safety profile, DOACs are more patient-friendly because dose monitoring and adjustment are not required and they have less clinically significant food and drug interactions (7).

In 2019, the results of a small exploratory randomized clinical trial on the safety and efficacy of dabigatran for the treatment of CVT (RESPECT-CVT) were published (8). While not powered to detect statistically significant differences, the data from RESPECT-CVT suggested a similar efficacy and safety of dabigatran compared to warfarin (a VKA). Data from other, mostly retrospective, studies appear to confirm the results from RESPECT-CVT and as a result, DOACs are increasingly being used to treat patients with CVT (9, 10). However, large-scale international prospective studies with data from a real-world setting have not been performed. The primary aim of the DOAC-CVT study is to evaluate the efficacy and safety of DOACs compared to VKAs for treatment of CVT in a real-world setting.

## 2. Methods and analysis

### 2.1. Study design and patient population

DOAC-CVT is an international, prospective, phase IV, comparative observational cohort study. Because of its observational design, the choice of oral anticoagulant type, dosage, and treatment duration are at the discretion of the treating physicians and patients’ preference.

Consecutive patients with CVT are recruited by participating centers. Patients are eligible for study participation if they meet the following criteria:

– Written informed consent by the patient or patient’s representative if required by local law;
– Age 18 years or older at the time of CVT diagnosis;
– CVT diagnosis radiologically confirmed by CT-venography, MRI, or catheter angiography;
– Oral anticoagulant treatment (DOAC or VKA) started within 30 days of CVT diagnosis. Patients may be initially treated with parenteral anticoagulants before starting oral anticoagulants.

Exclusion criteria of the study are:

– Patients who are already on anticoagulants at the time of CVT diagnosis;
– Patients with absolute contra-indications for DOACs, including one of the following:
– Pregnancy or lactation (post-partum women are eligible if they do not give breast-feeding);
– Mechanical heart valve;
– Severe renal insufficiency (defined as an estimated Glomerular Filtration Rate [eGFR] <15 ml/min);
– Severe liver disease resulting in clinically relevant coagulopathy.

Participating centers are asked to keep a recruitment log of all eligible patients. If a patient is not included in the study, the reason for exclusion is recorded in the log. In order to guard the generalizability of the study, no participating center can include more than 50 patients in the study. If a center reaches the mark of 50 participants, patient enrollment in that center will be halted.

### 2.2. Ethical aspects

The study is being conducted in accordance with the Declaration of Helsinki and the guidelines for Good Clinical Practice. The medical ethical review committee of Amsterdam UMC assessed that the study does not fall within the scope of the Medical Research Involving Human Subjects Act (in Dutch: wet medisch-wetenschappelijk onderzoek met mensen [WMO]) and provided a waiver for formal approval. All participating centers obtained local ethics approval for study participation if required by institutional regulations and applicable laws.

Eligible patients are informed about the study by the local investigators. In accordance with the European Union General Data Protection Regulation (GDPR), the patients or patients’ legal representatives provide written informed consent for the use of their pseudonymized data before enrollment in the study.

### 2.3. Study procedures

Pseudonymized data are collected in the secure online study database by the local investigators. All study data are collected as part of routine medical care. Data are collected at baseline, 3 months, 6 months, and 12 months after CVT diagnosis. Follow-up visits can be either face-to-face or remote consultations. Data on follow-up imaging for the assessment of venous recanalization are collected at 6 months after diagnosis. Detailed data on adverse events during follow-up are registered in the study database. In case of an adverse event, investigators are requested to create a report in which they can provide detailed information about the event. The report includes a summary of the adverse event and all relevant anonymized source documentation, such as hospital correspondence, any laboratory reports, imaging data, and treatment details at the time of the event. The study coordinators shall ensure that all reports on adverse events contain complete documentation and will be blinded for endpoint adjudication.

### 2.4. Definitions and outcomes

The primary objective of the study is to assess the safety and efficacy of DOACs versus VKAs in patients with CVT. The primary endpoint is the composite of symptomatic recurrent VTEs and major bleeding events after 6 months of follow-up. This is the same endpoint as used in RESPECT-CVT (8), facilitating a direct comparison between the two studies. Recurrent VTE is defined as one of the following: cerebral venous thrombosis, deep venous thrombosis of any limb, pulmonary embolism, and splanchnic vein, jugular, caval, renal, or catheter-related thrombosis. Major bleeding events are defined according to the criteria of the International Society on Thrombosis and Haemostasis (Table 1).

**Table 1:** Assessment of bleeding events.

Major Bleeding^*^
Symptomatic presentation and:
Fatal bleeding, and/or
Symptomatic bleeding in a critical area or organ, such as intracranial, intraspinal, intraocular, retroperitoneal, intra-articular or pericardial, or intramuscular with compartment syndrome, and/or
Bleeding causing a fall in hemoglobin level of 20 g/L (1.24 mmol/L) or more, or leading to transfusion of two or more units of whole blood or red cells.
Clinically relevant, non-major bleeding
Any sign or symptom of hemorrhage (e.g., more bleeding than would be expected for a clinical circumstance, including bleeding found by imaging alone) that does not fit the criteria for the ISTH definition of major bleeding but does meet at least one of the following criteria:
Requiring medical intervention by a healthcare professional;
Leading to hospitalization, prolongation of hospitalization or increased level of care;
Prompting a face-to-face evaluation (i.e., not just a telephone or electronic communication).

* Based on the definition of the International Society on Thrombosis and Haemostasis (ISTH) (11).

The following secondary endpoints are determined at 3, 6, and 12 months after CVT diagnosis:

– All-cause mortality;
– Symptomatic recurrent VTE rate;
– Major bleeding rate (Table 1) (11);
– Clinically relevant non-major bleeding rate (Table 1) (12);
– Arterial thrombotic event rate;
– Modified Rankin Scale score;
– Oral anticoagulant crossover rate and reasons for crossover.

The cerebral venous recanalization rate will be assessed if imaging is performed as part of routine clinical care. The imaging study is eligible if performed using an MRI, MR-venography, or CT-venography between 3 and 9 months after the CVT diagnosis. Recanalization will be scored according to the classification system described by Aguiar de Sousa et al. (13) (Table 2). For MRI, recanalization should be assessed by combining the data from MR-venography and conventional MR sequences, including paramagnetic-sensitive MR sequences (GRE-T2* or SWI), MR-venography, and T1-3D after gadolinium injection, if available.

**Table 2:** Classification of cerebral venous recanalization.

Definition of recanalization (scored per affected sinus or vein)^*^	
Complete recanalization	Restoration of blood flow in the entire sinus/vein. Narrowing of the lumen may be present, but must everywhere in the sinus/vein be less than 25% of the estimated normal diameter of the sinus/vein.
Partial recanalization	Restoration of blood flow in the entire sinus/vein, but with narrowing(s) of the venous lumen of more than 25% of the estimated normal diameter of the sinus/vein.
No recanalization/persistent occlusion	Fully interrupted blood flow in any part of the sinus/vein.

* Scored according to the classification system described by Aguiar de Sousa et al. (13).

All reported symptomatic recurrent VTEs, major bleedings, clinically relevant non-major bleedings, arterial thrombotic events, and deaths will be assessed by an adjudication committee. The members of the Adjudication Committee are listed in Supplementary Table 1. All relevant anonymized information about the adverse event, including clinical information, diagnostic test results, imaging slices, and local imaging reports, will be sent to the adjudication committee for evaluation. The adjudication committee will be blinded for the type of oral anticoagulant treatment used. Therefore, all information about DOAC or VKA use, including international normalized ratio (INR) values, will be removed from the documents. Investigators are asked to upload the relevant correspondence about adverse events in the original language. An English search engine translation will be provided by the study coordinators. Members of the Adjudication Committee are fluent in Dutch, English, French, German, Italian, Portuguese, and Spanish. The adjudication committee will use a standardized report form to assess all events (Supplementary File 2).

### 2.5. Sample size

We designed DOAC-CVT to be a pragmatic phase IV study. We aim to include at least 500 patients during the course of the study. Given the low event rate of the primary endpoint (estimated to be less than 3% in RESPECT-CVT), one would need to recruit more than 2000 patients to be able to prove non-inferiority of DOACs compared to VKAs (8). Because of the rarity of CVT (2), we estimated that it would not be feasible to recruit such a large number of patients within a reasonable study period. We therefore aim to recruit the maximum number of patients in a relatively short period of time through the International Cerebral Venous Thrombosis Consortium (14). Based on our previous collaborations, we estimate to recruit at least 500 patients in the three-year recruitment period. We expect a 3:2 ratio in DOAC:VKA use, resulting in approximately 300 included patients who are treated with DOACs and 200 treated with VKAs.

### 2.6. Benefits and risks assessment

Since we only collect routine medical care data, there is no additional burden for study participants and they are not at any risk. The study is strictly observational and we specifically do not impose any intervention or additional diagnostic tests. The decision of a patient to participate in the study does not have any consequences for the patient’s treatment. Study participants may withdraw their consent to participate in the study at any time without expressing any reason.

### 2.7. Statistical analysis plan

Analyses will be conducted according to the intentionto-treat principle. Patients will be grouped based on the first oral anticoagulant that was started (DOAC or VKA). Baseline characteristics will be presented for both groups (patients who were initially treated with DOACs and patients treated with VKAs). Counts and proportions will be provided for categorical data. Continuous data will be presented using means and standard deviations (SD) for normally distributed data and medians and interquartile ranges for non-normally distributed data. Any missing data on confounders will be imputed using multiple imputation.

#### 2.7.1. Analysis of the primary endpoint

**Figure 1:**
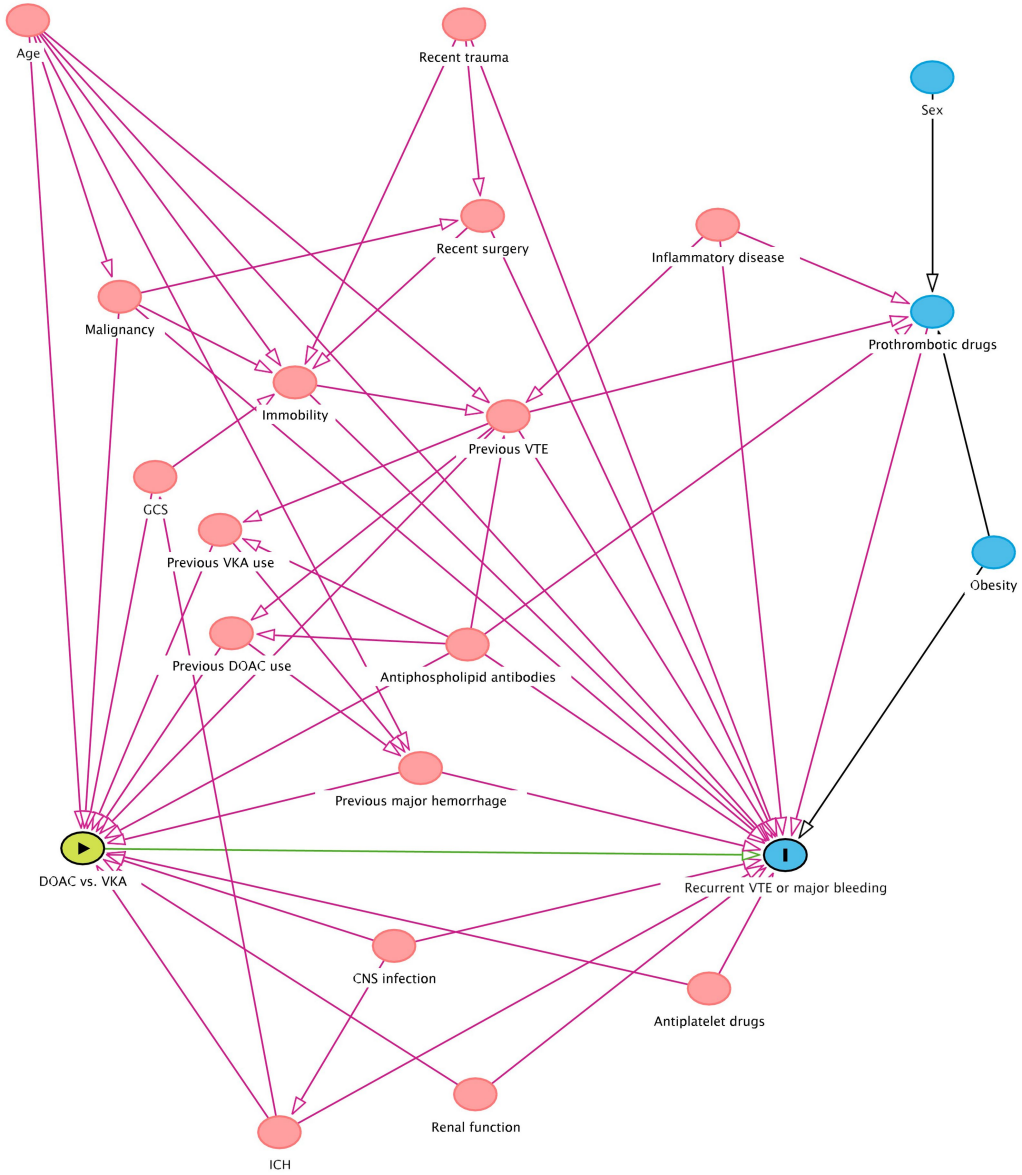
Directed Acyclic Graph depicting factors influencing anticoagulant treatment choice and risk of recurrent venous thrombotic event or major bleeding. CNS = central nervous system; DOAC = direct oral anticoagulant; GCS = Glasgow coma scale; ICH = intracerebral hemorrhage; VKA = vitamin K antagonist; VTE = venous thromboembolism. The green oval with the arrow represents the exposure, the blue oval with the vertical line represents the outcome, the other blue ovals represent the ancestors of outcome, and the pink ovals represent the ancestors of both exposure and outcome. The green line is the causal path, the purple lines the biasing paths.

We will use propensity score inverse probability treatment weighting to calculate an adjusted odds ratio for the primary outcome. Based on the direct acyclic graph (Figure 1), the following confounders will be used to compute the propensity score:

- Age;
- Baseline renal function;
- Cancer (defined as currently under treatment or diagnosed within 6 months prior to CVT diagnosis);
- Central nervous system (CNS) infection concurrent with the index CVT;
- Concomitant antiplatelet use at start of oral anticoagulant treatment;
- Country of inclusion’s income group as classified by The World Bank (15);
- Glasgow Coma Scale score at hospital presentation;
- Intracranial hemorrhage (ICH) before start of oral anticoagulant treatment;
- Known antiphospholipid syndrome (APS), or presence of antiphospholipid antibodies at start of oral anticoagulant treatment;
- Previous major bleeding prior to the index CVT (according to ISTH criteria [Table 1]);
- Previous VTE.

We will analyze the balance of confounders between both treatment groups after propensity score inverse probability weighting. *A last observation carried forward* approach will be used if the 6- or 12-month follow-up data are missing.

#### 2.7.2. Sensitivity analyses for the primary endpoint

In addition to the main analysis of the primary endpoint, we will perform four sensitivity analyses for the primary endpoint. Firstly, we will perform a survival analysis of the primary endpoint using the inverse probability weighting from the main analysis. Patients will be censored at the time of anticoagulant-switch or at the last follow-up moment (after 3, 6, or 12 months). Secondly, we will provide unadjusted analyses. Thirdly, we will repeat the analysis using a worstcase scenario approach i.e., using the assumption that all patients with missing outcome data have suffered a primary endpoint event. Lastly, we will perform a descriptive on-treatment analysis.

#### 2.7.3. Secondary study outcomes

All secondary outcomes will be analyzed following the same methods as used for the primary endpoint. Confounders to be included in each propensity score calculation are detailed in Supplementary Figures 1–5.

#### 2.7.4. Subgroup analysis

We will report all primary and secondary outcomes stratified by type of DOAC in an exploratory subgroup analysis if the number of cases is sufficient. In addition, we will perform a subgroup analysis for patients who were diagnosed with APS compared to patients who do not have APS. No formal statistical tests will be performed for these subgroup analyses.

## 3. Discussion

The aim of the DOAC-CVT study is to add relevant information on the efficacy and safety of DOACs for treatment of CVT and prevention of new VTEs in a real-world setting. This study will be the first international large-scale prospective study to investigate treatment with DOACs in patients with CVT. If the study provides evidence that DOACs are effective in preventing recurrent thrombosis and safe to use with regard to bleeding events, this will support their use as an alternative to VKAs in the treatment of CVT in clinical practice. At this moment, DOACs are not yet recommended in the European Stroke Organisation and American Heart Association/American Stroke Association guidelines for the treatment of CVT (4, 5).

The DOAC-CVT study is designed as a non-randomized cohort study. Because of the rarity of CVT and the low event rate of thrombotic recurrences and bleeding events (2, 8), it would be unrealistic to recruit a sufficient number of patients for a randomized controlled trial within the time frame of the study. In addition, an experimental study design would have made it more difficult for centers worldwide to participate in the study due to more extensive regulatory requirements and high administrative costs. Therefore, we have chosen the pragmatic, prospective, observational study design, which is probably the highest feasible level-of-evidence study design to address this clinical question. By balancing covariates in the two treatment-arms using propensity score inverse probability treatment weighting, we aim to increase the validity of the results.

In order to truly reflect a real-world setting and increase the generalizability of the study results, we specifically aim to include patients with various ethnic backgrounds from a large number of countries across a large geographical area. In addition, we aim to include a diverse group of patients by only excluding patients who are already using anticoagulants at the time of CVT diagnosis and patients with absolute contraindications for DOACs.

All events of interest that occur during follow-up, such as recurrent thrombotic events, bleeding events, and deaths, will be adjudicated by an Adjudication Committee. We believe this adjudication is important to ensure a high level of certainty about the nature of the adverse events. As the expected incidence of the primary outcome events, i.e., thrombotic recurrences and major bleeding events after CVT, is low, it will be vital to confirm and classify these events accurately. To prevent any treatment bias when assessing the adverse events, the Adjudication Committee will be blinded for the type of oral anticoagulant the patient used at the time of the outcome event. The independent adjudication of outcome events will distinguish the DOAC-CVT study from previous large cohort studies (10).

This international multi-center cohort study will provide new evidence on the safety and efficacy of DOACs for treatment of CVT in a real-world setting. The results of this study, in conjunction with the previous exploratory randomized controlled trial, retrospective studies, and the recently completed SECRET trial (15) will guide physicians in the shared decision process of selecting the best oral anticoagulant type for patients with CVT in the future.

## 4. Conclusion

The DOAC-CVT study will provide real-world data on the comparative efficacy and safety of DOACs versus VKAs for the treatment of CVT. If DOACs are found to be effective in preventing recurrent thrombosis and safe to use with regard to bleeding events, they can be widely implemented as an oral anticoagulant treatment option for CVT besides VKAs.

## 5. Ethics and dissemination

### 5.1. Processing of data

No directly identifying data, such as name, address, or date of birth, are collected for the purpose of this study. All patients are assigned a unique study ID upon inclusion. The identification key linking this study ID to the patient’s personal data is stored by the local investigator at the participating hospital. The identification key will not be shared with the coordinating hospital.

Amsterdam UMC functions as the data controller for this study. Amsterdam UMC has a data processor agreement with the electronic data capture system (Castor EDC, Ciwit B.V., Amsterdam, The Netherlands). The participating centers are responsible for processing, storing, and transferring the patient data in compliance with local laws and institutional rules. Participating centers signed a Data Sharing Agreement with Amsterdam UMC. The study data will be archived for at least 15 years after inclusion of the last patient before it will be destroyed.

## 6. Trial status

**Figure 2:**
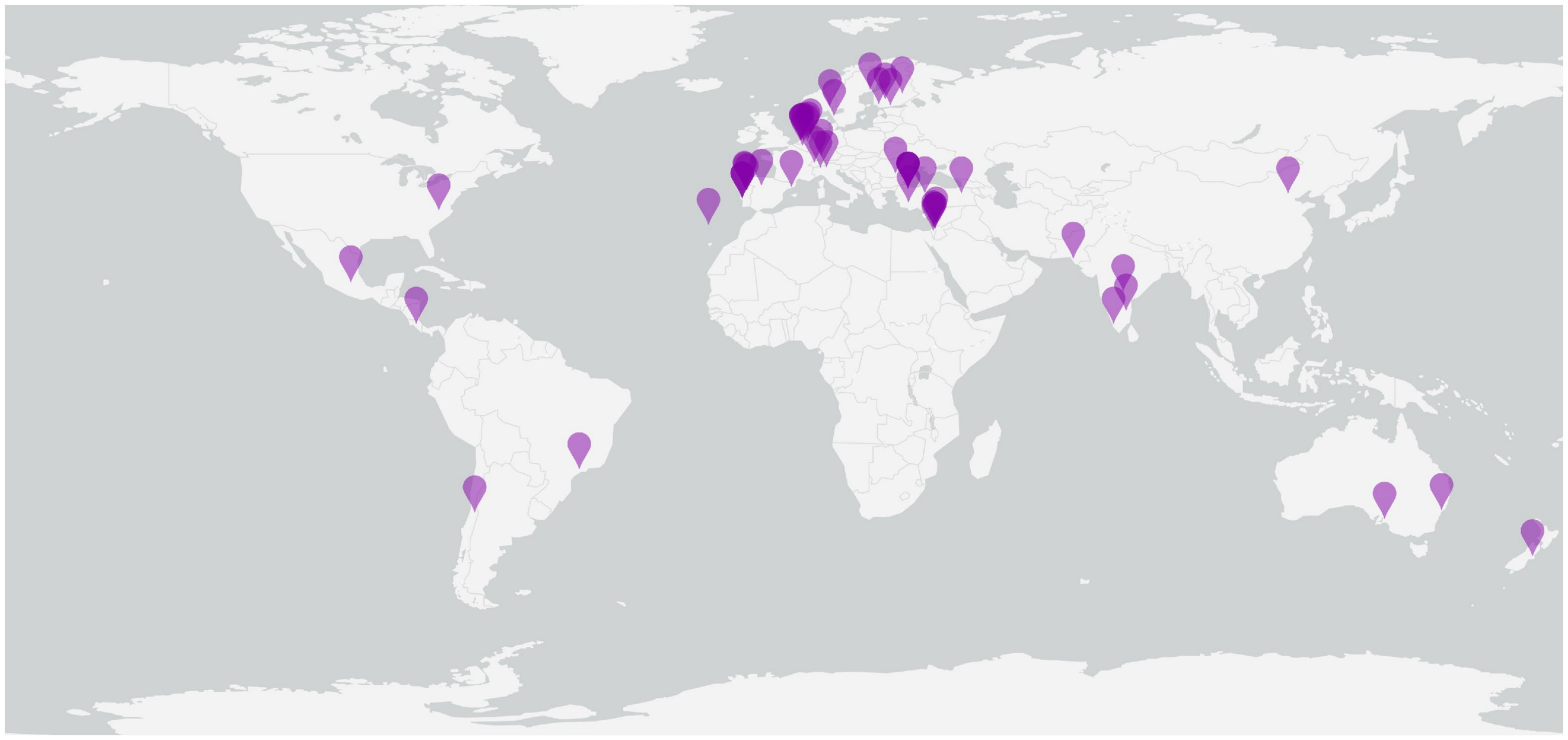
Centers active in the DOAC-CVT study. Purple pins represent centers that are actively recruiting patients in the DOAC-CVT study.

Patients are currently being recruited for participation in the DOAC-CVT study. Patient enrollment has started in January 2021. By 22 June 2023, 477 patients from 54 centers in 23 countries have been included in the study (Figure 2). We are still recruiting new centers for study participation. Investigators who are interested in participating in the DOAC-CVT study may contact the corresponding author for more information.

## Ethics statement

This study involving humans was approved by the medical ethical review committee of Amsterdam UMC. This study is conducted in accordance with the local legislation and institutional requirements. The participants provide their written informed consent to participate in this study.

## Author contributions

MSK, KK, JP, TT, JF, and JC conceived the study, were involved in protocol development and gaining ethical approval. AM and MSK wrote the first draft of the manuscript. All authors contributed to the article and approved the submitted version.

## The DOAC-CVT study group

An overview of all DOAC-CVT investigators is provided in Supplementary Table 2.

## Funding

The author(s) declare financial support was received for the research, authorship, and/or publication of this article. The study is funded by the Netherlands Thrombosis Foundation (in Dutch: Trombosestichting) with project number 2020_02.

## Conflict of interest

DA reports travel support from Boehringer Ingelheim, speaker fees from Bayer, and Advisory Board participation for AstraZeneca; MH reports grants from Swiss National Science Foundation, SITEM Support Funds and Swiss Heart Foundation, all outside the submitted work; TK has received educational meeting cost assistance from Boehringer Ingelheim; RL reports fees paid to his institution for consultancy by Boehringer Ingelheim, Genentech, Ischemaview, Medtronic and Medpass; SP has received research support from BMS/Pfizer, Boehringer-Ingelheim, Daiichi Sankyo, European Union, German Federal Joint Committee Innovation Fund, German Federal Ministry of Education and Research, Helena Laboratories, and Werfen as well as speakers’ honoraria/consulting fees from Alexion, AstraZeneca, Bayer, Boehringer-Ingelheim, BMS/Pfizer, Daiichi Sankyo, Portola, and Werfen (all outside of the submitted work); MA reports personal fees from AstraZeneca, Bayer, Bristol Myers Squibb, Covidien, Daiichi Sankyo, Medtronic, Novartis, Pfizer, and Amgen; TT has received personal fees from Bayer, Boehringer Ingelheim, Bristol Myers Squibb, Inventiva, and Portola Pharma; JF has received personal fees from Boehringer Ingelheim, Bayer, and Daiichi Sankyo as well as grants from Bayer; JC has received grants paid to his institution from Boehringer Ingelheim and Bayer, and payments paid to his institution for data safety monitoring board participation by Bayer.

The remaining authors declare that the research was conducted in the absence of any commercial or financial relationships that could be construed as a potential conflict of interest.

## Publisher’s note

All claims expressed in this article are solely those of the authors and do not necessarily represent those of their affiliated organizations, or those of the publisher, the editors and the reviewers. Any product that may be evaluated in this article, or claim that may be made by its manufacturer, is not guaranteed or endorsed by the publisher.

## Figures and Tables

**Table:** 

CNS	Central nervous system
CT	Computerized tomography
CVT	Cerebral venous thrombosis
DOAC	Direct oral anticoagulants
eGFR	Estimated glomerular filtration rate
GCS	Glasgow Coma Scale
GDPR	General Data Protection Regulation
GRE-T2*	Gradient echo T2*-weighted imaging
ICH	Intracranial hemorrhage
INR	International normalized ratio
ISTH	International Society on Thrombosis and Haemostasis
MRI	Magnetic Resonance Imaging
mRS	Modified Rankin Scale
SD	Standard deviation
SWI	Susceptibility weighted imaging
UMC	University Medical Centers
VKA	Vitamin K antagonists
VTE	Venous thromboembolism
WMO	Wet medisch-wetenschappelijk onderzoek met mensen (Dutch law)
