# Corrigendum: Direct oral anticoagulants for the treatment of cerebral venous thrombosis – a protocol of an international phase IV study

**DOI:** 10.3389/fneur.2024.1481563

**Published:** 2024-09-23

**Authors:** Anita van de Munckhof, Mayte Sánchez van Kammen, Katarzyna Krzywicka, Sanjith Aaron, Diana Aguiar de Sousa, Florina Antochi, Antonio Arauz, Miguel A. Barboza, Adriana B. Conforto, Francesco Dentali, Daniel Galdames Contreras, Xunming Ji, Katarina Jood, Mirjam R. Heldner, María Hernández-Pérez, Wayneho Kam, Timothy J. Kleinig, Espen S. Kristoffersen, Ronen R. Leker, Robin Lemmens, Sven Poli, Nilüfer Yeşilot, Mohammad Wasay, Teddy Y. Wu, Marcel Arnold, Lia Lucas-Neto, Saskia Middeldorp, Jukka Putaala, Turgut Tatlisumak, José M. Ferro, Jonathan M. Coutinho

**Affiliations:** ^1^Department of Neurology, Amsterdam UMC, Location University of Amsterdam, Amsterdam, Netherlands; ^2^Department of Neurology, Christian Medical College, Vellore, India; ^3^Department of Neurology, Stroke Center, Centro Hospitalar Universitário de Lisboa Central, Lisbon, Portugal; ^4^Department of Neurology, Spitalul Universitar de Urgentă Bucureşti, Bucharest, Romania; ^5^Department of Neurology, National Institute of Neurology and Neurosurgery, Mexico City, Mexico; ^6^Department of Neurology, Rafael Angel Calderon Guardia Hospital, San José, Costa Rica; ^7^Department of Neurology, Hospital das Clínicas da Faculdade de Medicina da Universidade de São Paulo, São Paulo, Brazil; ^8^Department of Neurology, Asst Sette Laghi, Varese, Italy; ^9^Stroke Unit, Hospital Clínico de la Universidad de Chile, Santiago, Chile; ^10^Department of Neurology, Xuanwu Hospital, Capital Medical University, Beijing, China; ^11^Department of Neurology, Sahlgrenska University Hospital, Gothenburg, Sweden; ^12^Department of Clinical Neuroscience, Institute of Neuroscience and Physiology, Sahlgrenska Academy at University of Gothenburg, Gothenburg, Sweden; ^13^Department of Neurology, Inselspital, University Hospital and University of Bern, Bern, Switzerland; ^14^Department of Neurology, Hospital Germans Trias i Pujol, Badalona, Spain; ^15^Department of Neurology, Duke University Hospital, Durham, NC, United States; ^16^Department of Neurology, Royal Adelaide Hospital, Adelaide, SA, Australia; ^17^Department of Neurology, Akershus University Hospital, Nordbyhagen, Norway; ^18^Department of Neurology, Hadassah – Hebrew University Medical Center, Jerusalem, Israel; ^19^Department of Neurology, UZ Leuven, Leuven, Belgium; ^20^Department of Neurology, Tübingen University Hospital, Tübingen, Germany; ^21^Department of Neurology, Istanbul Tip Fakültesi, Istanbul, Turkey; ^22^Department of Neurology, Aga Khan University, Karachi, Pakistan; ^23^Department of Neurology, Christchurch Hospital, Christchurch, New Zealand; ^24^Department of Neuroradiology, Centro Hospitalar Universitário Lisboa Norte, Lisbon, Portugal; ^25^Department of Internal Medicine, Radboud University Medical Center, Nijmegen, Netherlands; ^26^Department of Neurology, Helsinki University Hospital and University of Helsinki, Helsinki, Finland; ^27^Centro de Estudos Egas Moniz, Faculdade de Medicina, Universidade de Lisboa, Lisbon, Portugal

**Keywords:** cerebral venous thrombosis, anticoagulants, DOAC, vitamin K antagonist, treatment

Two corrections have been made to the Statistical Analysis Plan as previously published in Section **2. Methods and analysis**, subsection “*2.7. Statistical analysis plan*.” First, given the large number of participating centers and the expected low incidence of the primary endpoint, adjusting for center of inclusion in the outcome model is not considered feasible. Instead, we added country of inclusion's income group as classified by The World Bank (15) to the list of confounders, which will be used to model the propensity score.

Second, we will not use multiple imputation for missing outcome data, but only for missing data on confounders. Given that most events occur in the first period after diagnosis, (10) the last observation carried forward approach will be used if the 6- and 12-month follow-up data are missing. To assess the influence of this approach, we will perform an additional sensitivity analysis conducting a worst-case scenario approach i.e., using the assumption that all patients with missing outcome data would have suffered a primary endpoint event.

Additionally, for clarification, the on-treatment analysis as described in Section “*2.7.2. Sensitivity analyses for the primary endpoint*” will be descriptive only.

The DOAC-CVT Executive Committee decided on these changes on July 15, 2024, prior to closure of the database.

**Section 2. Methods and analysis**, subsection “*2.7. Statistical analysis plan*” will now read:

## 2.7. Statistical analysis plan

Analyses will be conducted according to the intention-to-treat principle. Patients will be grouped based on the first oral anticoagulant that was started (DOAC or VKA). Baseline characteristics will be presented for both groups (patients who were initially treated with DOACs and patients treated with VKAs). Counts and proportions will be provided for categorical data. Continuous data will be presented using means and standard deviations (SD) for normally distributed data and medians and interquartile ranges for non-normally distributed data. Any missing data on confounders will be imputed using multiple imputation.

### 2.7.1. Analysis of the primary endpoint

We will use propensity score inverse probability treatment weighting to calculate an adjusted odds ratio for the primary outcome. Based on the direct acyclic graph (Figure 1), the following confounders will be used to compute the propensity score:

- Age;
- Baseline renal function;
- Cancer (defined as currently under treatment or diagnosed within 6 months prior to CVT diagnosis);
- Central nervous system (CNS) infection concurrent with the index CVT;
- Concomitant antiplatelet use at start of oral anticoagulant treatment;
- Country of inclusion's income group as classified by The World Bank (15);
- Glasgow Coma Scale at hospital presentation;
- Intracranial hemorrhage (ICH) before start of oral anticoagulant treatment;
- Known antiphospholipid syndrome (APS), or presence of antiphospholipid antibodies at start of oral anticoagulant treatment;
- Previous major bleeding prior to the index CVT (according to ISTH criteria [Table 1]);
- Previous VTE.

We will analyze the balance of confounders between both treatment groups after propensity score inverse probability weighting. A *last observation carried forward* approach will be used if the 6- or 12-month follow-up data are missing.

### 2.7.2. Sensitivity analyses for the primary endpoint

In addition to the main analysis of the primary endpoint, we will perform four sensitivity analyses for the primary endpoint. Firstly, we will perform a survival analysis of the primary endpoint using the inverse probability weighting from the main analysis. Patients will be censored at the time of anticoagulant-switch or at the last follow-up moment (after 3, 6, or 12 months). Secondly, we will provide unadjusted analyses. Thirdly, we will repeat the analysis using a worst-case scenario approach i.e., using the assumption that all patients with missing outcome data have suffered a primary endpoint event. Lastly, we will perform a descriptive on-treatment analysis.

### 2.7.3. Secondary study outcomes

All secondary outcomes will be analyzed following the same methods as used for the primary endpoint. Confounders to be included in each propensity score calculation are detailed in Supplementary Figures 1–5.

### 2.7.4. Subgroup analysis

We will report all primary and secondary outcomes stratified by type of DOAC in an exploratory subgroup analysis if the number of cases is sufficient. In addition, we will perform a subgroup analysis for patients who were diagnosed with APS compared to patients who do not have APS. No formal statistical comparisons will be performed for these subgroup analyses.

The authors apologize for these errors and state that this does not change the scientific conclusions of the article in any way. The original article has been updated.

