# Supplementary material for: Direct oral anticoagulants for the treatment of cerebral venous thrombosis – a protocol of an international phase IV study
Source: Front Neurol. 2023 Sep 14;14:1251581. doi: 10.3389/fneur.2023.1251581 (PMC10539579; doi:10.3389/fneur.2023.1251581)
Supplement: Supplementary file 1 [file Data_Sheet_1.PDF]

Supplementary Material

Direct Oral Anticoagulants for the Treatment of Cerebral Venous Thrombosis (DOAC-CVT) – A Protocol of an International Phase IV Study

Table of contents:

Supplementary Table 1.	Overview of DOAC-CVT study committee members.	2
Supplementary Table 2.	The DOAC-CVT Study Group	3
Supplementary Figure 1.	Directed Acyclic Graph depicting factors influencing anticoagulant treatment choice and risk of recurrent venous thrombotic events.	4
Supplementary Figure 2.	Directed Acyclic Graph depicting factors influencing anticoagulant treatment choice and risk of major or clinically relevant non-major bleeding.	5
Supplementary Figure 3.	Directed Acyclic Graph depicting factors influencing anticoagulant treatment choice and risk of death or dependency.	6
Supplementary Figure 4.	Directed Acyclic Graph depicting factors influencing anticoagulant treatment choice and risk of arterial thrombotic events.	7
Supplementary Figure 5.	Directed Acyclic Graph depicting factors influencing anticoagulant treatment choice and cerebral venous recanalization.	8

Supplementary Table 1. Overview of DOAC-CVT study committee members.

Executive Committee	J.M. Coutinho
	J.M. Ferro
	J. Putaala
	T. Tatlisumak
Steering Committee	S. Aaron
	D. Aguiar de Sousa
	F. Antochi
	A. Arauz
	M.A. Barboza
	A.B. Conforto
	F. Dentali
	D. Galdames Contreras
	X. Ji
	K. Jood
	M.R. Heldner
	M. Hernandez-Perez
	W. Kam
	T.J. Kleinig
	E.S. Kristoffersen
	R.R. Leker
	R. Lemmens
	S. Poli
	N. Yesilot
	M. Wasay
	T. Wu
Adjudication Committee	M. Arnold
	S. Middeldorp
	L. Neto

Supplementary Table 2. The DOAC-CVT Study Group

Jaime Alonso Maroto	Elias Johansson	Federica Rizzo
Tooba Arshad	Wilmar Jolink	Alejandro Rodríguez
Matilda Arvgård	Valtteri Julkunen	Bob Roozenbeek
Orli Barad	Hanna Kämppi	Marta Rubiera Del Fueyo
Anastassia Blanter	Anne-Mari Kantanen	Jori Ruuskanen
Noit Brown	Hala Kassem	Tzika Sacagiu
Christine Bruelisauer	Erin Kerr	Yara Safadi
Sebastiaan de Bruijn	Einat Kohn	Tomi Sarkanen
Nazlı Bülbül	Marianne Kormann	Adrian Scutelnic
Ilidia Carmezim	Nyika Kruyt	Joaquín Serena
Marta Carvalho	Tanja Kumpulainen	Mine Sezgin
María Castañón Apilanez	Mercedes de Lera Alfonso	Radi Shahien
Jennifer Cranefield	Erik Lindgren	Jussi Sipilä
Matin Daftari	Gert-Jan Luijckx	Maria Sofia Cotelli
Shaked Dahan	Evelyn Marcelis	Santiago Trillo
Alexandria d'Emden	Nicolas Martinez Majander	Anil Tuladhar
Jelle Demeestere	Lénea Maria Martins Porto	Boby Varkey Maramattom
Annemie Devroye	Joshua Mbroh	Merelijne Verschoof
Tamara Dofferhoff-Vermeulen	Joke de Meris	Eva Vister
Jiangang Duan	Murat Mert Atmaca	Ido van den Wijngaard
Esme Ekizoglu	Gert Messchendorp	Işıl Yazıcı Gençdal
Naziha El Ghannouti	Ipek Midi	Pauli Ylikotila
Alper Eren	Yael Mimoun	Julia Zeller
Nesrin Ergin	Cesar Minelli	Femke Zitman
Tiago Esteves Freitas	Ana Minerva Vazquez Quezadas	
Luis Fontão	Angel Miraclin	
Blanca Eulalia Fuentes Gimeno	Jeremy Molad	
Carlos Garcia Esperon	Francisco Moniche	
Ana Gomes	Y. Muralidhar Reddy	
Naama Goren	Laura Nevaharju Sarantis	
Ana Gouveia	Zoë Oberli	
Rosemary Green	David Orion	
Daniel Guisado Alonso	Ana Paiva Nunes	
Alina Hampshire Santibañez Nuñez	João Pedro de Seabra Marto	
Mine Hayriye Sorgun	Janika Pietilä	
Heleen den Hertog	Eva Ponjee	
Sini Hiltunen	Kathlein Pouza	
Secil Irmak	Inka Puhakka	
Terhi Ivanoff	Alden P. van Putten	
Pekka Jäkälä	Ayala Rabi	

Supplementary Figure 1. Directed Acyclic Graph depicting factors influencing anticoagulant treatment choice and risk of recurrent venous thrombotic events.

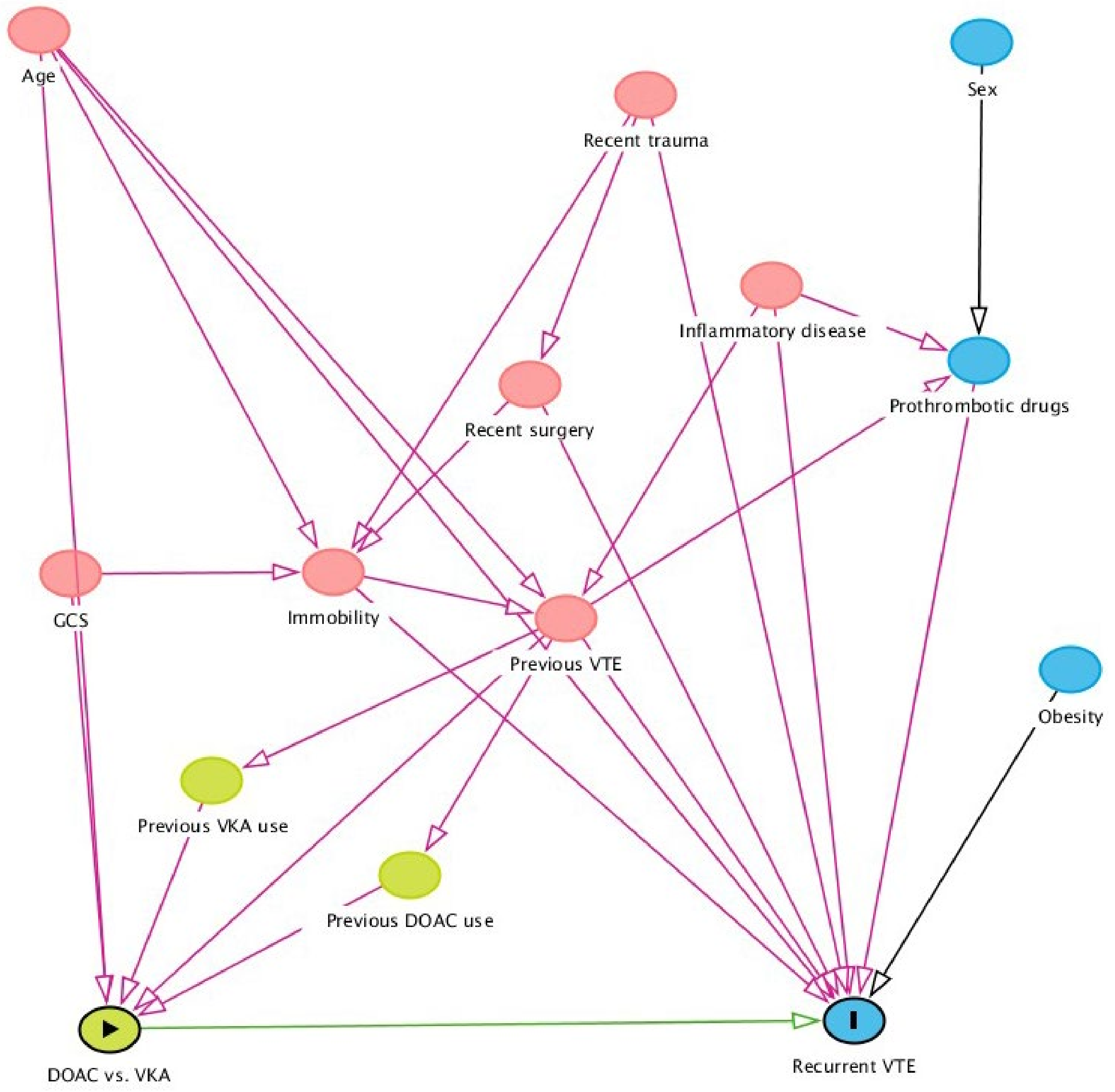

CVT = cerebral venous thrombosis; DOAC = direct oral anticoagulant; GCS = Glasgow Coma Scale; VKA = vitamin K antagonist; VTE = venous thromboembolism.

The green oval with the arrow represents the exposure, the blue oval with the vertical line represents the outcome, the other green ovals represent the ancestors of exposure, the blue ovals represent the ancestors of outcome, and the pink ovals represent the ancestors of both exposure and outcome. The green line is the causal path, the purple lines the biasing paths.

Confounders to be included in the propensity score calculation for outcome symptomatic recurrent VTE rate:

- Age at time of CVT diagnosis;
- GCS score at CVT diagnosis;
- Previous VTE.

Supplementary Figure 2. Directed Acyclic Graph depicting factors influencing anticoagulant treatment choice and risk of major or clinically relevant non-major bleeding.

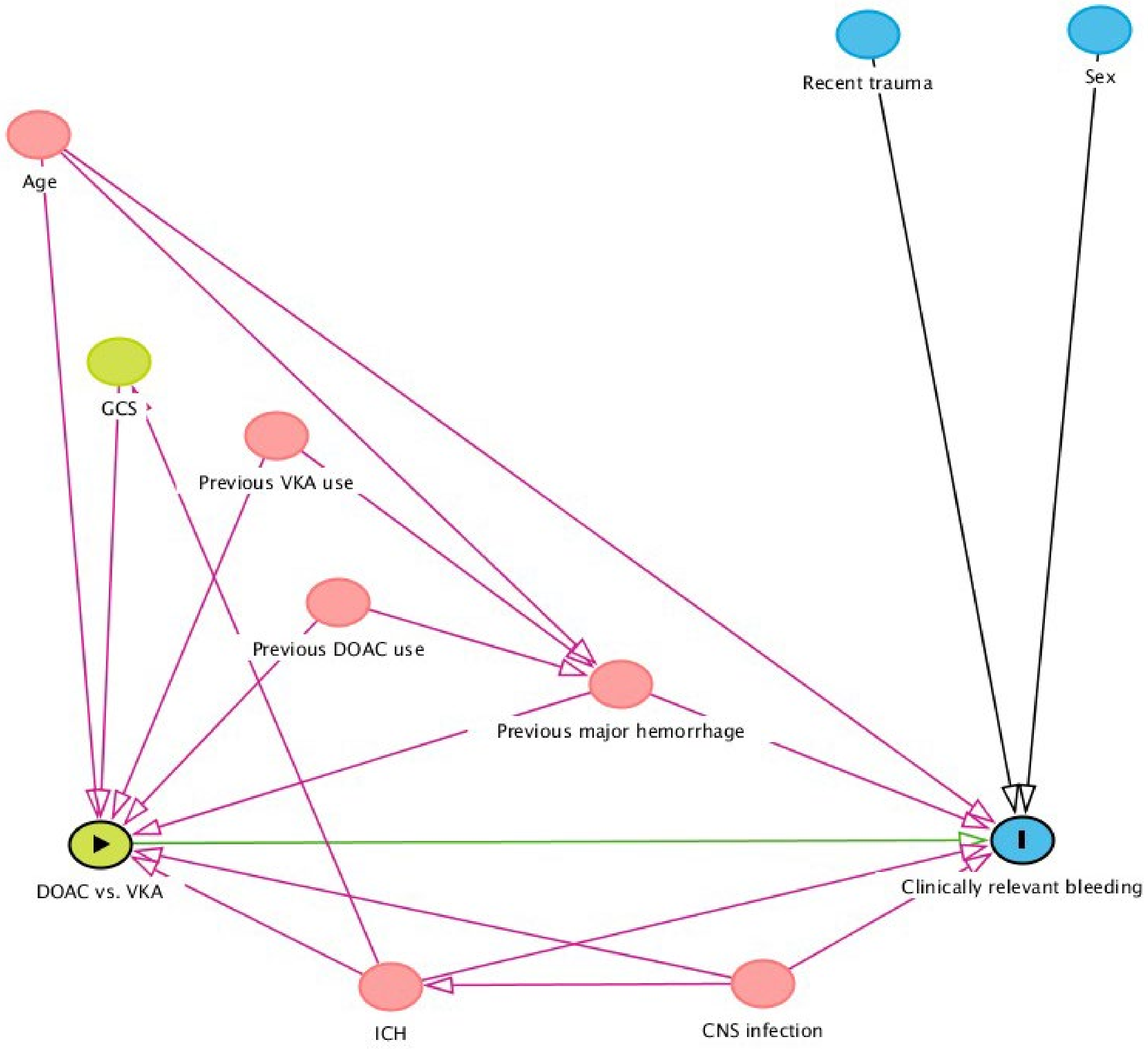

CNS = central nervous system; CVT = cerebral venous thrombosis; DOAC = direct oral anticoagulant; GCS = Glasgow Coma Scale; ICH = intracranial hemorrhage; VKA = vitamin K antagonist; VTE = venous thromboembolism.

The green oval with the arrow represents the exposure, the blue oval with the vertical line represents the outcome, the green oval represents the ancestor of exposure, the blue ovals represent the ancestors of outcome, and the pink ovals represent the ancestors of both exposure and outcome. The green line is the causal path, the purple lines the biasing paths.

Confounders to be included in the propensity score calculation for outcomes ‘Major Bleeding event’ and ‘Clinically Relevant, Non-Major Bleeding event’ (Table 1):

- Age at CVT diagnosis;
- CNS infection concurrent with the index CVT;
- ICH at CVT diagnosis, or after diagnosis but before start of oral anticoagulant treatment;
- Previous major bleeding prior to the index CVT (definition in Table 1).

Supplementary Figure 3. Directed Acyclic Graph depicting factors influencing anticoagulant treatment choice and risk of death or dependency.

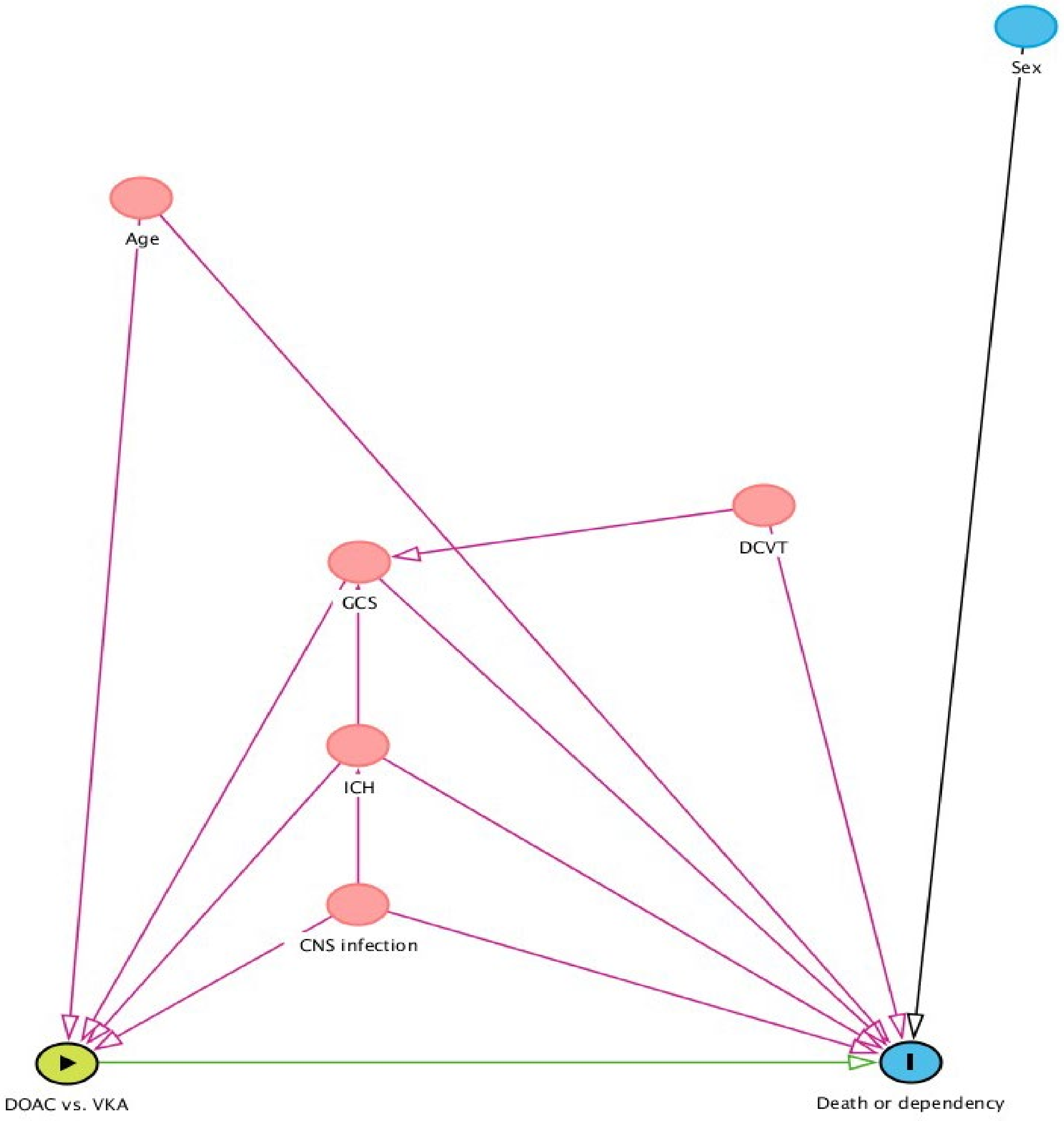

CNS = central nervous system; CVT = cerebral venous thrombosis; DCVT = cerebral venous thrombosis of the deep venous system; DOAC = direct oral anticoagulant; GCS = Glasgow Coma Scale; ICH = intracranial hemorrhage; VKA = vitamin K antagonist.

The green oval with the arrow represents the exposure, the blue oval with the vertical line represents the outcome, the other blue oval represents the ancestor of outcome, and the pink ovals represent the ancestors of both exposure and outcome. The green line is the causal path, the purple lines the biasing paths.

Confounders to be included in the propensity score calculation for outcomes all-cause mortality and modified Rankin Scale score:

- Age at CVT diagnosis;
- CNS infection concurrent with the index CVT;
- GCS score at CVT diagnosis;
- ICH before start of oral anticoagulant treatment.

Supplementary Figure 4. Directed Acyclic Graph depicting factors influencing anticoagulant treatment choice and risk of arterial thrombotic events.

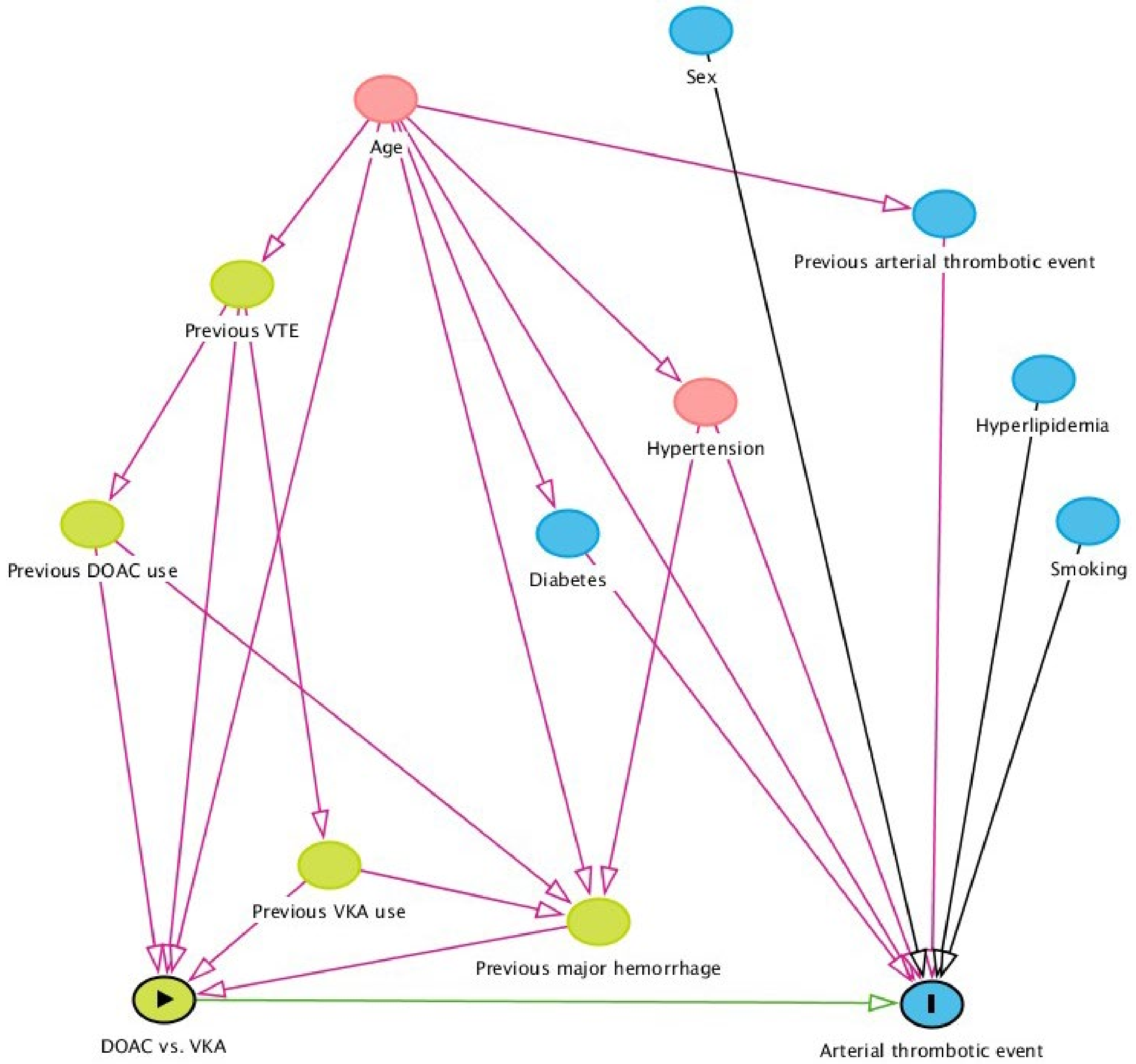

CVT = cerebral venous thrombosis; DOAC = direct oral anticoagulant; VKA = vitamin K antagonist; VTE = venous thromboembolism.

The green oval with the arrow represents the exposure, the blue oval with the vertical line represents the outcome, the other green ovals represent the ancestors of exposure, the blue ovals represent the ancestors of outcome, and the pink ovals represent the ancestors of both exposure and outcome. The green line is the causal path, the purple lines the biasing paths.

Confounders to be included in the propensity score calculation for outcome arterial thrombotic events:

- Age at CVT diagnosis;
- History of hypertension (as determined by treating physician).

Supplementary Figure 5. Directed Acyclic Graph depicting factors influencing anticoagulant treatment choice and cerebral venous recanalization.

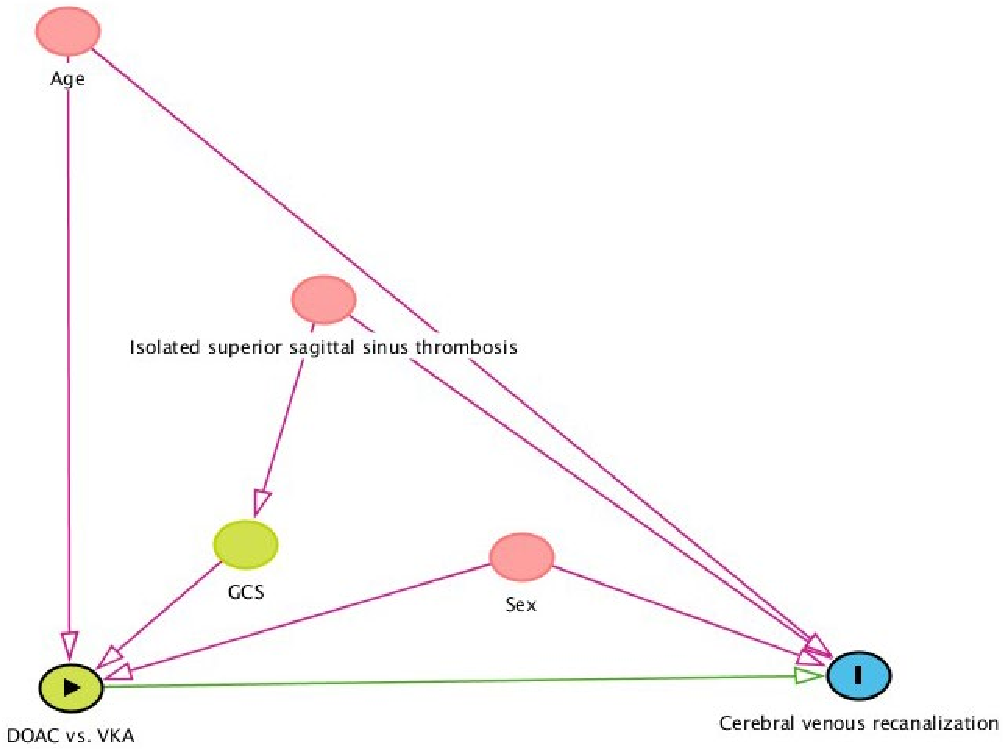

DOAC = direct oral anticoagulant; GCS = Glasgow Coma Scale; VKA = vitamin K antagonist.

The green oval with the arrow represents the exposure, the blue oval with the vertical line represents the outcome, the green oval represents the ancestor of exposure, and the pink ovals represent the ancestors of both exposure and outcome. The green line is the causal path, the purple lines the biasing paths.

Confounders to be included in the propensity score calculation for cerebral venous recanalization rate (definition in Table 2):

- Age at CVT diagnosis;
- Sex;
- Isolated superior sagittal sinus thrombosis at diagnosis.
