# Supplementary material for: Direct oral anticoagulants for the treatment of cerebral venous thrombosis – a protocol of an international phase IV study
Source: Front Neurol. 2023 Sep 14;14:1251581. doi: 10.3389/fneur.2023.1251581 (PMC10539579; doi:10.3389/fneur.2023.1251581)
Supplement: Supplementary file 2 [file Data_Sheet_2.PDF]

DOAC-CVT ADJUDICATION MANUAL

**Version**	1.1
**Date**	01 July 2023
**Project leader**	Dr. Jonathan Coutinho<br>Amsterdam UMC<br>Email: j.coutinho@amsterdamumc.nl
**Sponsor**	Amsterdam UMC<br>Meibergdreef 9<br>1105 AZ Amsterdam
**Subsidising party**	Trombosestichting Nederland<br>Dobbeweg 1a<br>2254 AG Voorschoten

Authors: Mayte Sánchez van Kammen, Katarzyna Krzywicka, Saskia Middeldorp, Jose M. Ferro, Turgut Tatlisumak, Jukka Putaala, Jonathan M. Coutinho

TABLE OF CONTENTS

0. INTRODUCTION……………………………………………………………………4
1. ENDPOINTS FOR ADJUDICATION……………………………………………4
2. DEFINITION OF ENDPOINTS…………..……………………………………..4
    2.1 Death…………………………………………………………………………...4
    2.2 New Venous Thromboembolism ……………………………………………...6
    2.3 Major Bleeding………………………………………………………………...7
    2.4 Clinically Relevant, Non Major Bleeding……………………………………..7
    2.5 Arterial Thrombotic Events…………………………..………………………..8
3. ADJUDICATION PACKAGE…………..………………………………………...8
    3.1 Patient profile…………………………………………………………………8
    3.2 Source documentation…………………………………………………………8
    3.3 Re-Adjudication Criteria and Process…………………………………………8
4. ADJUDICATION PROCESS…………………..…………………………………..9
4. ADJUDICATION COMMITTEE MEMBERS…….…………………………………..9
5. APPENDICES……………….…………………..……………………………………9

LIST OF ABBREVIATIONS

AC	Adjudication Committee
ATE	Arterial thromboembolism
CT	Computed tomography
CV	Cardiovascular
CVT	Cerebral venous thrombosis
DOAC	Direct oral anticoagulants
DVT	Deep vein thrombosis
VKA	Vitamin K antagonists
HF	Heart failure
ICH	Intracranial hemorrhage
ISTH	International Society on Thrombosis and Haemostasis
VTE	Venous thromboembolism
MI	Myocardial infarction
MRI	Magnetic resonance imaging
PE	Pulmonary embolism
SIRS	Systemic Inflammatory Response Syndrome

0. INTRODUCTION

The Adjudication Committee (AC) is an independent expert group who will be responsible for the central adjudication of events of major and clinically relevant bleeding, venous thromboembolism (VTE), and cause of death for the DOAC-CVT study. The adjudicators will be blinded to anticoagulant treatment and all additional information which could lead to reveal of the anticoagulant. The study started in 2021 and is expected to be active through 2024. Approximately 500 patients are planned to be enrolled in this study. It is estimated that approximately 25 potential events will be identified and adjudicated. Committee members will be blinded to oral anticoagulant use. The primary contact for AC members is the Principal Investigator. This Manual describes the process for adjudication of events.

1. ENDPOINTS FOR ADJUDICATION

- Death (cause)
- VTE
    - Deep Vein Thrombosis (DVT)
    - Pulmonary Embolism (PE)
    - Splanchnic Vein Thrombosis
    - New CVT
- Major Bleeding
- Clinically Relevant Non-Major Bleeding (CRNMB)
- Arterial thrombotic events (ATE)

2. DEFINITION OF ENDPOINTS

2.1 Death

Cause of death will be classified by the AC as due to a fatal VTE, fatal ATE, other cardiovascular, non-cardiovascular cause, or undetermined cause of death. The cause of death will be determined by the principal condition that caused death, not the immediate mode of death. AC members will review all available information and use their clinical expertise to adjudicate the cause of death. For fatal events the onset date of the event leading to death should be reported by the committee. If available, study sites should provide death certificates for patients who have died. However, if a death certificate is the only information available for review besides the patient profile in the study database, if another etiology appears to be more plausible or precise, the AC may decide to reject what is recorded on the death certificate as a cause of death.

2.1.1 Fatal VTE

Death due to fatal VTE includes death due to fatal pulmonary embolism and other VTE, including CVT.

2.1.2 Fatal ATE

Includes death due to death due to stroke, death resulting from an acute myocardial infarction and other ATE's.

2.1.3. Other Cardiovascular Death

This category includes all cardiovascular deaths not encompassed by 2.1.1 and 2.1.2, including sudden cardiac death, death due to heart failure, death due to cardiovascular (CV) procedures, death due to CV hemorrhage, and death due to other CV causes (e.g., peripheral arterial disease).

Note: Unless additional information suggests an alternate specific cause of death, if a patient is seen alive </= 24 hours of being found dead, cardiovascular death (namely sudden cardiac death) should be recorded. For patients who were not observed alive within 24 hours of death, undetermined cause of death should be recorded (e.g., a subject found dead in bed, but who had not been seen by family for several days).

2.1.4 Non-Cardiovascular Death
Non-cardiovascular death is defined as any death with a specific cause that is not thought to be of cardiovascular nature. Examples of non-cardiovascular death are: pulmonary causes (excluding pulmonary embolism), renal causes, gastrointestinal causes (excluding splanchnic ischemic or venous thrombosis, hepatobiliary causes, pancreatic causes, infection (including sepsis), inflammatory (e.g., systemic inflammatory response syndrome), immune (including autoimmune - may include environmental anaphylaxis, e.g., food allergies), hemorrhage that is neither cardiovascular bleeding or stroke, non-CV procedure or surgery, trauma, suicide, non-prescription drug reaction or overdose, prescription drug reaction or overdose (may include anaphylaxis), neurological (non-cardiovascular), malignancy (i.e., new malignancy, worsening of prior malignancy) or other (should be specified).

2.1.5 Undetermined Cause of Death
Undetermined cause of death refers to a death not attributable to a specific category. Inability to classify the cause of death may be due to lack of information (e.g., the only available information is "patient died") or when there is insufficient supporting information or detail to assign the cause of death. In general, most deaths can be classifiable as CV or non-CV, and the use of this category of death should be minimal.

2.2 New Symptomatic Venous Thromboembolism (VTE)
New VTE will be defined as symptomatic new CVT, DVT of any limb, PE, splanchnic vein, jugular, caval, renal, ovarian, catheter-related thrombosis, or other venous thrombosis after start of oral anticoagulant treatment. Superficial vein thrombosis is not considered a symptomatic VTE.

2.2.1 New Cerebral Venous Thrombosis (CVT)
CVT refers to occlusion of venous channels in the cranial cavity, including dural venous thrombosis, cortical vein thrombosis and deep CVT. Defined as new neurological symptoms or worsening of previous symptoms with new CVT on neuroimaging. Enlargement of a previous sinus or vein thrombosis without new clinical symptoms is not considered as a new CVT. CVT must be confirmed by AC review of relevant imaging slices and reports of at least one of the following:

- MRI + MR venography (recommended)
- CT + CT venography
- MRI or CT in combination with catheter angiography
- Autopsy

2.2.2 Deep Vein Thrombosis (DVT) of any limb

Defined as symptomatic DVT of any limb. DVT must be confirmed by AC review of relevant imaging slices and reports of at least one of the following:

- Abnormal compression ultrasonography
- An intraluminal filling defect on venography (any modality)
- Autopsy

2.2.3 Pulmonary Embolism (PE)

New symptomatic unilateral or bilateral PE confirmed by definitive objective ancillary investigations according to international standards. PE must be confirmed by AC review of relevant imaging slices and reports of at least one of the following:

- Ventilation-perfusion (V-Q) lung scan
- Pulmonary angiography (any modality?)
- CT pulmonary angiography
- Inconclusive spiral CT, pulmonary angiography or lung scintigraphy with demonstration of DVT in the lower extremities by compression ultrasonography or venography
- Autopsy

2.2.4 Splanchnic Vein Thrombosis

Defined as the symptomatic presence of endoluminal material or absence of flow in the extrahepatic portal veins, splenic vein or mesenteric vein. Splanchnic vein thrombosis must be confirmed by the AC by review of relevant imaging slices and reports of at least one of the following:

- Duplex-Doppler Ultrasound
- Contrast-enhanced CT scan
- MRI
- Autopsy

2.2.5 Jugular Vein Thrombosis

Defined as symptomatic thrombosis of the jugular vein, not extending into the cerebral venous sinuses and not related to use of intravenous catheters. Jugular vein thrombosis must be confirmed by the AC by review of relevant imaging slices and reports of at least one of the following:

- Duplex-Doppler Ultrasound
- Contrast-enhanced CT scan
- MRI
- Catheter angiography
- Autopsy

2.2.6 Caval Vein Thrombosis

Defined as symptomatic thrombosis of the caval vein not related to use of intravenous catheters. Caval vein thrombosis must be confirmed by the AC by review of relevant imaging slices and reports of at least one of the following:

- Duplex-Doppler Ultrasound
- Contrast-enhanced CT scan

- MRI
- Catheter angiography
- Autopsy

2.2.7 Renal Vein Thrombosis

Defined as symptomatic thrombosis of the renal vein. Renal vein thrombosis must be confirmed by the AC by review of relevant imaging slices and reports of at least one of the following:

- Contrast-enhanced CT scan
- MRI
- Catheter angiography
- Autopsy

2.2.8 Catheter-related Thrombosis

Defined as symptomatic thrombosis of the deep veins related to the use of intravenous catheters. Catheter-related thrombosis must be confirmed by AC review of relevant imaging slices and reports of at least one of the following:

- Duplex-Doppler Ultrasound
- MRI + MR venography
- Catheter-based contrast venography
- Autopsy

2.3 Major Bleeding

Major bleeding will be defined as meeting at least one of the following criteria according to the International Society on Thrombosis and Haemostasis (ISTH) definition:

Symptomatic presentation and
- Fatal bleeding, and/or
- Symptomatic bleeding in a critical area or organ, such as intracranial, intraspinal, intraocular, retroperitoneal, intra-articular or pericardial, or intramuscular with compartment syndrome, and/or
- Bleeding causing a fall in hemoglobin level of 20 g/L (1.24 mmol/L) or more, or leading to transfusion of two or more units of whole blood or red cells

Major bleedings will also be assessed to determine the site of the bleeding:

a) Intracranial (ICH) – comprises the subtypes of intracerebral bleedings, subdural bleedings, epidural bleedings, and subarachnoid bleedings;
b) Gastrointestinal – any bleeding that occurs in the upper or lower gastrointestinal tract;
c) Other bleeding location – any bleeding location other than intracranial or gastrointestinal.

2.4 Clinically Relevant, Non-Major Bleeding (CRNMB)
A CRNMB is a clinically overt bleeding that does not meet the criteria for a major bleeding, but prompts a clinical response, in that it leads to at least one of the following:

Any sign or symptom of hemorrhage (e.g., more bleeding than would be expected for a clinical circumstance, including bleeding found by imaging alone) that does not fit the criteria for the ISTH definition of major bleeding but does meet at least one of the following criteria:
- Requiring medical intervention by a healthcare professional
- Leading to hospitalization, prolongation of hospitalization or increased level of care
- Prompting a face to face (i.e., not just a telephone or electronic communication) evaluation

2.5 Arterial Thrombotic Event
Arterial thrombotic events are defined as myocardial infarction, ischemic stroke, TIA, acute limb ischemia, or other arterial thrombotic event.

2.5.1 Myocardial infarction
Defined as clinical evidence of acute myocardial ischemia and detection of a rise of cardiac troponin values with at least one value above the 99th percentile, and at least one of the following:

- ●Symptoms of myocardial ischemia
- ●New ischemic electrocardiographic (ECG) changes
- ●Development of pathological Q waves
- ●Imaging evidence of new loss of viable myocardium or new regional wall motion abnormality in a pattern consistent with an ischemic etiology
- ●Identification of a coronary thrombus by angiography or autopsy

2.5.2 Ischemic stroke
Defined as an episode of neurological dysfunction caused by cerebral, spinal or retinal infarction, based on:

- Imaging evidence of ischemic injury in a defined vascular distribution, or
- Clinical evidence of ischemic injury based on symptoms persisting >24 hours or until death, and other etiologies excluded

Preferably confirmed by AC review of imaging and /or reports of a head CT and/or MRI.

2.5.3 TIA
Defined as an episode of neurological dysfunction caused by cerebral, spinal or retinal ischemia, with clinical symptoms lasting less than 24 hours, and without evidence of acute infarction. Preferably confirmed by AC review of imaging and /or reports of a head CT and/or MRI.

2.5.4 Acute limb ischemia
Defined as a quickly developing (<2 weeks) or sudden decrease in limb perfusion, usually producing new or worsening symptoms or signs, and often threatening limb viability. May be clinically diagnosed (including assessment of the ankle-brachial index bilaterally) or diagnosed by a combination of clinical assessment and vascular imaging (such as CT

angiography or catheter-based angiography). In case of phlegmasia cerulea dolens, i.e., when perfusion is compromised by venous thrombosis and congestion, this will be adjudicated as DVT.

2.5.5 Other arterial thrombotic event

Ad diagnosed by the local treating physician, and confirmed by AC review of correspondence and relevant imaging.

3. ADJUDICATION PACKAGE

All case adjudication packages will be prepared and blinded by the coordinating investigator before the Adjudication Committee meetings. A search engine translation and original version of all anonymized source documents will be provided. Each blinded case package consists of a cover sheet, patient profile, and any available source documentation. The case cover form can be found in Appendix A.

3.1 Patient profile

The patient profile will consist of the following data:

- Demographics
- Medical and Surgical History
- Narrative of the clinical course of the event
- CVT history, including information on imaging modality, and thrombophilia information if available
- Trigger Events (Fatal Events, VTE, Bleeding)
- Concomitant medications (except anticoagulation type)
- Physical exam/vitals
- D-dimer and other relevant laboratory test results (excluding INR, anti-Xa values)

3.2 Source documentation

If requested by the AC, the following anonymized source documentation will be requested (may be provided in original language):

- Correspondence relevant to the event (e.g. hospital admission and discharge letters)
- Imaging reports relevant to the event
- Imaging slices relevant to the event
- Laboratory results relevant to the event (e.g. hemoglobin levels)

3.3 Re-Adjudication Criteria and Process

Events will be re-adjudicated if clinically relevant new information becomes available or information previously documented for a case was incorrectly reported for data provided to the AC. If new/changed information would predict a possible change in decision the case will be sent for re-adjudication.

4. ADJUDICATION PROCESS

All suspected events will be adjudicated during a plenary meeting of the AC. During the meeting, the AC will complete the applicable Adjudication Form for each suspected event. The Adjudication Forms can be found in Appendix B. Depending on the number of suspected events, one or two meetings will be held during the final year of study recruitment (or before if requested by the Steering Committee). The meetings may be held in-person or as video calls.

5. ADJUDICATION COMMITTEE MEMBERS

Prof. Saskia Middeldorp, vascular internist, chair<br>*Department of Internal Medicine, Radboud Institute for Health Sciences, Radboud University Medical Centre, Nijmegen, Netherlands*
Dr. Lia Neto, neuroradiologist<br>*Hospital de Santa Maria, Lisbon, Portugal*
Prof. Marcel Arnold, neurologist<br>*Bern University Hospital, Bern, Switzerland*

If deemed necessary, consultation of an independent cardiologist or radiologist will be requested.

6. APPENDICES

Appendix A. Case Cover Form

DOAC-CVT Study Adjudication Package

Patient number: ....

Date of Adjudication: ....

Type of review
☐Initial
☐Re-adjudication: Date of original adjudication:

Type of event
☐Fatal event
☐Bleeding
☐VTE
☐ATE

Event start date: ....
Event start time: ....

List of what is included in the package
☐Images: specify location: .... and type: ....;
☐Source document, specify type:...;
☐Patient profile, date: ....

Appendix B. Adjudication Form

Patient number: .... **Date of event:** ....

Date of Adjudication: ....

Fatal Event (complete if applicable)

Classify the Event

☐ Fatal VTE
☐ Fatal ATE
☐ Other cardiovascular death
☐ Non-cardiovascular death
☐ Undetermined Cause of Death
  ☐ Insufficient Supporting Information or detail
  ☐ Lack of Information, specify:…………………………………………………
  ☐ Other, specify:……………………………………………… ……………..

Bleeding events (complete if applicable)

Major Bleeding

Was the event a major bleeding?
☐ Yes
☐ No
☐ Event is non-assessable, provide reason:
  ☐ Poor quality of data provided
  ☐ Lack of data, specify: …………………………………………………………
  ☐ Other, specify:…………………………...……………………………..

Classify site of major bleeding:
☐ Intracranial hemorrhage
☐ Gastrointestinal bleeding
☐ Other bleeding location, specify:……………………………………………………

Clinically Relevant Non-Major Bleeding

Was the event a clinically relevant non-major bleeding?
☐ Yes
☐ No
☐ Event is non-assessable, provide reason:
  ☐ Poor quality of data provided
  ☐ Lack of data, specify: …………………………………………………………
  ☐ Other, specify:………………………………… ……………………………..

Appendix B. Adjudication Form (continued)

Patient number: .... **Date of event:** ....

Date of Adjudication: ....

VTE Event (complete if applicable)

Was the event a Venous Thromboembolism?

☐ Yes
☐ No
☐ Event is non-assessable, provide reason:
    ☐ Poor quality of data provided
    ☐ Lack of data, specify: …………………………………………………………
    ☐ Other, specify:………………………………… ………………………………..

Classify the event:

☐ Cerebral Venous or Dural Sinus Thrombosis
☐ Deep Vein Thrombosis
☐ Pulmonary Embolism
☐ Deep Vein Thrombosis plus Pulmonary Embolism
☐ Splanchnic Vein Thrombosis
☐ Jugular Vein Thrombosis
☐ Caval Vein Thrombosis
☐ Renal Vein Thrombosis
☐ Catheter-related Thrombosis
    ☐ Specify location:……………………………………………………………
☐ Other, specify:………………………………… ……………………………..………

Arterial thrombotic event (complete if applicable)

Was the event an arterial thrombotic event?

☐ Yes
☐ No
☐ Event is non-assessable, provide reason:
    ☐ Poor quality of data provided
    ☐ Lack of data, specify: …………………………………………………………
    ☐ Other, specify:………………………………… ………………………………..

Classify the event:

☐ Myocardial Infarction
☐ Ischemic Stroke
☐ Transient Ischemic Attack
☐ Acute Limb Ischemia
☐ Other, specify:………………………………… ……………………………..………
